# Fabrication of eco-friendly graphene-based superhydrophobic coating on steel substrate and its corrosion resistance, chemical and mechanical stability

**DOI:** 10.1038/s41598-022-14353-0

**Published:** 2022-06-22

**Authors:** M. E. Mohamed, A. Ezzat, A. M. Abdel-Gaber

**Affiliations:** Chemistry Department, Faculty of Science, Alexandria University, Alexandria, Egypt

**Keywords:** Chemical engineering, Electrochemistry, Materials chemistry, Surface chemistry

## Abstract

Superhydrophobic coatings were successfully fabricated on steel substrates using potentiostatic electrodeposition of Ni and Ni-graphene, Ni-G, coatings followed by immersion in an ethanolic solution of stearic acid, SA. Rice straw, an environmentally friendly biomass resource, was used to synthesize high-quality graphene. The Raman spectra proved the high quality of the produced graphene. The Fourier transform infrared spectroscopy, FTIR, results showed that the Ni coating grafted with stearic acid, Ni-SA, and the Ni-G composite grafted with stearic acid, Ni-G-SA, were successfully deposited on the steel substrate. The scanning electron microscope, SEM, results showed that the prepared superhydrophobic coatings exhibit micro-nano structures. The wettability results revealed that the values of contact angles, CAs, for Ni-SA and Ni-G-SA coatings are 155.7° and 161.4°, while the values of sliding angles, SAs, for both coatings are 4.0° and 1.0°, respectively. The corrosion resistance, chemical stability, and mechanical abrasion resistance of the Ni-G-SA coating were found to be greater than those of the Ni-SA coating.

## Introduction

Extremely non-wettable surfaces are one of nature's most interesting aspects. Due to the extremely low stickiness of liquid drops on natural non-wettable surfaces forms a sphere shape and instantly roll off the surface^1^. The extremely water repellent surfaces which exhibit a contact angle greater than 150° are famously known as superhydrophobic surfaces^2^. Superhydrophobic surfaces have aroused a lot of interest because of their importance in fundamental science and industrial applications. Superhydrophobic surfaces have a variety of applications such as oil–water separation^3^, anti-icing^4^, self-cleaning^5^, corrosion resistance^6^, drag reduction^7^, sensors^8^, solar cells^9^, biomedical^10^, microfluidic devices^11^, and antifouling technologies^12^. Different superhydrophobic coatings with remarkable water repellency can be created by enhancing the surface roughness, which is the first requirement for superhydrophobicity and lowering the surface energy, which is the second requirement for superhydrophobicity^13^. Creating a surface with these characteristics can be difficult, especially when environmental and consumer safety issues are present. Historically, the used low surface energy material is perfluorinated compounds, including fluoro silanes or fluorocarbon molecules, due to their ultralow surface energy (≈ 10 mJ m^−2^)^14^. However, using such long-chained fluorocarbons has been proven to be very toxic and possess negative environmental outcomes such as persistence, biomagnification, and bioaccumulation^2,14–17^. Hence, there is a need to develop low-cost, eco-friendly methods and environmentally friendly materials in fabricating superhydrophobic surfaces^18^. Ilker S. Bayer recently published a review that looked at numerous viable approaches for fabricating superhydrophobic and even superoleophobic coatings using environmentally friendly technologies and biodegradable components such as waxes, lipids, proteins, and cellulose^14^. This review explains, evaluates, and examines such advancements and their performance in comparison to traditional approaches.

For the preparation of superhydrophobic coatings, various methods have been proposed, including immersion^19^, electrospinning^20^, electrodeposition^6^, layer self-assembly^21^, plasma etching^4^, chemical vapour deposition^22^, electrochemical anodic oxidation^23^, phase separation^24^, dipping^25^, spraying^2^, and sol–gel methods^26^. Electrodeposition is an excellent technique for constructing artificial superhydrophobic surfaces because of its low-temperature process, clean, low cost, simplicity, and controllable nanostructure^6^.

Steel has a wide range of applications due to its high mechanical strength and relatively low price. However, it has high electrochemical and chemical activities to corrosion attack^27,28^. Generally, corrosion is regarded as one of our societies' most serious issues with economic and safety implications^29–31^. Many protective techniques can be used to protect steel surfaces^28,32^; one of the most significant is the fabrication of superhydrophobic coatings, which significantly increase steel corrosion resistance^33,34^.

However, the major disadvantages of poor mechanical durability and mechanical instability restrict the practical applications of superhydrophobic surfaces^35,36^. To be used in industrial applications, superhydrophobic surfaces must increase their mechanical abrasion resistance and chemical stability.

Graphene is a honeycomb-like carbon allotrope with a two-dimensional structure. Graphene is one of the most remarkable nanomaterials since it is not only the thinnest carbon-based nanostructure but also one of the most robust. Graphene is a good material for coatings, especially anti-corrosion coatings, because of its strength, single atomic layer thickness, chemical inertness, and impermeability to most gases^37,38^. Chemical reduction of graphene oxide, exfoliation of graphite, epitaxial growth on silicon carbide, and chemical vapour deposition (CVD) are the four main methods for graphene manufacturing^39^. Unfortunately, the majority of these approaches are time-consuming and involve the use of hazardous chemicals and gases. Due to time and production quality constraints, some of them are not suitable for industrial mass production^40^. Many scientists are currently working on developing green synthesis methods for graphene manufacturing^39–41^. Rice straw, an environmentally friendly biomass resource, is used to make graphene in this study. Rice straw is the most widely produced agricultural material in the world, with around 120 million tons produced each year^39^. In recent years, most farmers have opted for the simplest method of production: burning rice straw; nevertheless, this has severe consequences, such as air pollution, especially as the number of burnings grows. The detrimental impact on the environment is reduced by converting this waste into more valuable materials like graphene.

This study aims to fabricate superhydrophobic graphene-based coating at the steel surface. Stearic acid is used as a low surface energy material which is an environmentally friendly and cheap compound^42^. An environmentally eco-friendly method was used to synthesize high-quality graphene from a biomass resource, rice straw. The wettability, mechanical and chemical stability, and corrosion resistance properties were measured for the prepared superhydrophobic coatings in an aqueous solution of 0.5 M NaCl.

## Experimental

### Materials

A steel plate with dimensions of 2.0 cm × 1.0 cm × 0.1 cm was used as a substrate. The rice straw was purchased from a local market. Anhydrous ethanol, nickel chloride hexahydrate, nickel sulfate, boric acid, sodium hydroxide, potassium hydroxide, and sulfuric acid of analytical grade were used.

### Graphene synthesis from rice straw

This synthesis consists of three steps: pre-treatment, chemical activation, and post-treatment. The pre-treatment stage includes washing the rice straw many times to remove all debris, followed by burning it for approximately 15 min at 250 °C to form the rice straw ash, RSA. The chemical activation process includes mixing RSA (4 g) and KOH (20 g) in a crucible; then, the crucible is covered by ceramic wool. The crucible was placed into a larger crucible. The space between the two crucibles was filled with RSA, which served as a barrier to prevent the sample inside the smaller crucible from oxidizing. In a muffle furnace, the sample was annealed at 700 °C for 2.5 h. In the post-treatment step, the sample was washed many times with distilled water to remove excess KOH before drying for 24 h at 100 °C.

### Superhydrophobic coating preparation

The steel substrate was mechanically polished with emery paper of various grades before electrodeposition, beginning with coarse one (grade 300) and progressing to the finest in stages (800 grade). The substrate was then degreased in a soap solution for 10 min, then activated by immersion in 2.0 M H_2_SO_4_ for one minute, then rinsed with distilled water and ethanol before being directly immersed in the electrodeposition bath. The electrodeposition parameters for the fabrication of Ni coating and Ni-graphene, Ni-G, coating on the steel substrate are depicted in Table 1. A platinum sheet with the same dimensions as the steel substrate was utilized as an anode and was separated with a 2.0 cm gap from the steel substrate, the cathode. The Ni and Ni-G coatings were rinsed with distilled water and then dried at room temperature for a day. The dry coated Ni and Ni-G coatings substrates were immersed in ethanolic solutions of 0.01 M stearic acid (SA) for 0.25 h and then dried at room temperature. The prepared Ni coating grafted by stearic acid, Ni-SA, and the Ni-G coating grafted by stearic acid, Ni-G-SA, were subjected to various characterization and evaluation methodologies.

**Table 1:** Bath compositions and operating conditions for electrodeposition of Ni and Ni-graphene coating on the steel substrate.

Factor		Level	
		Ni	Ni-G
(Nickel ion source)	NiCl_2_⋅6H_2_O	40 gL^−1^	
	NiSO_4_	176 gL^−1^	
(Buffer the pH)	H_3_BO_3_	60 gL^−1^	
Deposition time		6.0 min	
Optimal deposition potential		11.0 V	
Graphene		0.0 gL^−1^	0.2 gL^−1^

### Surface characterization

A scanning electron microscope, SEM (model JSM-200 IT, JEOL), was used to examine the surface topography of the generated superhydrophobic coatings. The surface chemical composition was analyzed using the Fourier transform infrared spectrophotometer (model: Bruker Tensor 37 FTIR). The reported spectra are in the 4000–400 cm^−1^. X-ray diffraction investigation was performed with monochromatic Cu K radiation (= 0.154056 nm) using an X-ray diffractometer (Bruker D2 phaser). Raman spectra of graphene were obtained using spectrometer (Senttera-Broker) equipped with 532 nm wavelength laser. Water contact angle (CA) and sliding angle (SA) were estimated with 5 µL water droplets using an optical contact angle goniometer (Rame-hart CA instrument, model 190-F2). The CAs and SAs values presented are the averages of two measurements carried out at different substrate locations.

### Mechanical abrasion

The scratch test was utilized to analyze the mechanical abrasion properties of the produced superhydrophobic coatings. The prepared superhydrophobic coating samples were placed on 800 mesh sandpaper, and 3.0 kPa pressure was applied to them. The prepared superhydrophobic sample was moved horizontally, and the CA and SA were measured for each 3.0 cm abrasion length. The reported mechanical abrasion resistance is the average of values taken on two different samples.

### Chemical stability

A water droplet of different pH values (pH = 1–13) was placed on the prepared superhydrophobic coatings, and the CAs and SAs were determined for each pH^43^. Sulfuric acid and sodium hydroxide were used to control the water droplet pH. The reported CAs and SAs are the average of two tests performed on the sample's surface at different places.

### Corrosion tests

The electrochemical measurements were performed with a three-electrode cell on an ACM frequency response analyzer (UK). A graphite rod and an Ag/AgCl electrode were served as the counter and reference electrodes, respectively. The bare steel and steel coated by superhydrophobic Ni-SA and Ni-G-SA coatings were used as working electrodes. An epoxy layer was applied to the working electrodes, leaving 1 cm^2^ exposed to the testing solution. The working electrode was placed in a cell containing 0.5 M NaCl solution that was opened to the atmosphere at room temperature and left for 20 min before electrochemical measurements to reach the equilibrium potential. The frequency range of the electrochemical impedance spectroscopy (EIS) measurements was 0.1 ≤ f ≤ 1.0 × 10^4^ with an applied potential signal amplitude of 10 mV around the equilibrium potential. The polarization measurements were conducted at a 30 mV/min scan rate using a potential range of ± 250 mV around the equilibrium potential. Experiments were double-checked to ensure that the measurements were accurate and the results were within 2% error.

## Results and discussion

### Raman spectra

**Figure 1:**
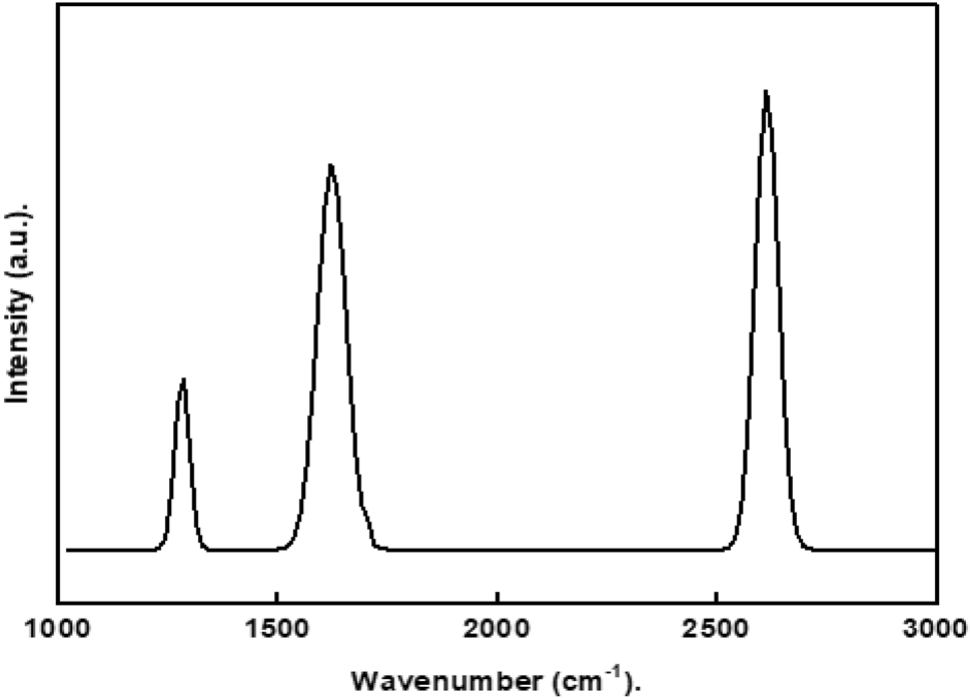
Raman spectra of the prepared graphene.

Raman scattering is a powerful non-destructive technique that is highly useful in examining the ordered and disordered crystallographic structure^44^. Figure 1 depicts the Raman spectrum of graphene. The D peak at 1286 cm^−1^ is produced by the breathing mode of the sp^2^ atoms, which is active in the presence of defects and impurities in graphene^45^, whereas the G peak at 1621 cm^−1^ is generated by the E_2g_ phonon of sp^2^ hybridized carbon atoms. Graphene also has a high 2D peak, around 2612 cm^−1^. The 2D peak, on the other hand, is well known to be the second-order of the D peak. The number of layers has a significant influence on the shape, position, and strength of this peak relative to the D band. Therefore, a sharp 2D peak proved that graphene was successfully synthesized^46^.

### Fourier transform infrared spectroscopy, FTIR, results

**Figure 2:**
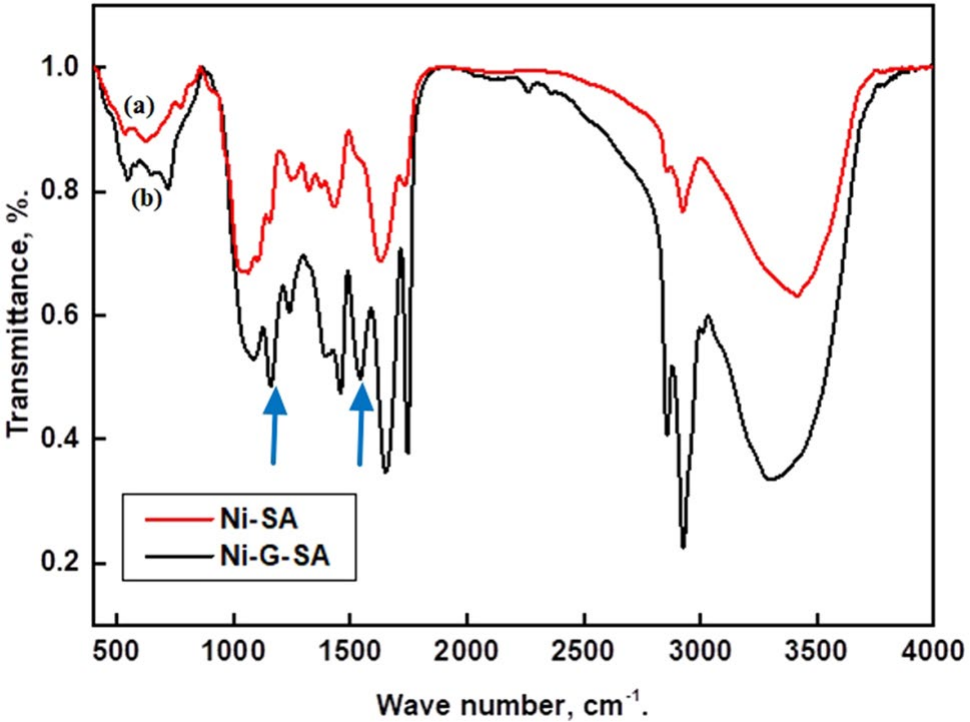
FTIR spectra of steel coated by (**a**) Ni-SA coating and (**b**) Ni-G-SA coating.

Figure 2 shows the FTIR spectra of steel coated with Ni-SA and Ni-G-SA. The spectrum demonstrates that graphene modifies the produced nickel-stearic acid coating. The appearance of a peak at 1543 cm^−1^ in the Ni-G-SA coating is consistent with C=C double bonds in the polycyclic aromatic graphene ring. Furthermore, C–O stretching is responsible for the band at 1154 cm^−1^. Otherwise, comparable bands appear for Ni-SA and Ni-G-SA coatings. The peaks at 3300 cm^−1^ correspond to O–H bonds stretching vibrations of stearic acid, complemented by the C–OH band at 1158 cm^−1^ due to the hydroxyl groups^47^. The two peaks at 2927 cm^−1^ and 2857 cm^−1^ are assigned to –CH_2_– asymmetry and symmetry vibration^48^. The peak at 1745 cm^−1^ is attributed to the C=O stretch. The peak at 1653 cm^−1^ corresponds to O–H bonds bending vibrations. The two peaks at 1457 and 1391 cm^−1^ correspond to the bending vibration of C–H^49^. At 1083 cm^−1^, the C–O–C band characteristic of epoxy appears^50^. The peak at 716 cm^−1^ corresponds to Ni(OH)_2_^33^.

### SEM and wettability results

**Figure 3:**
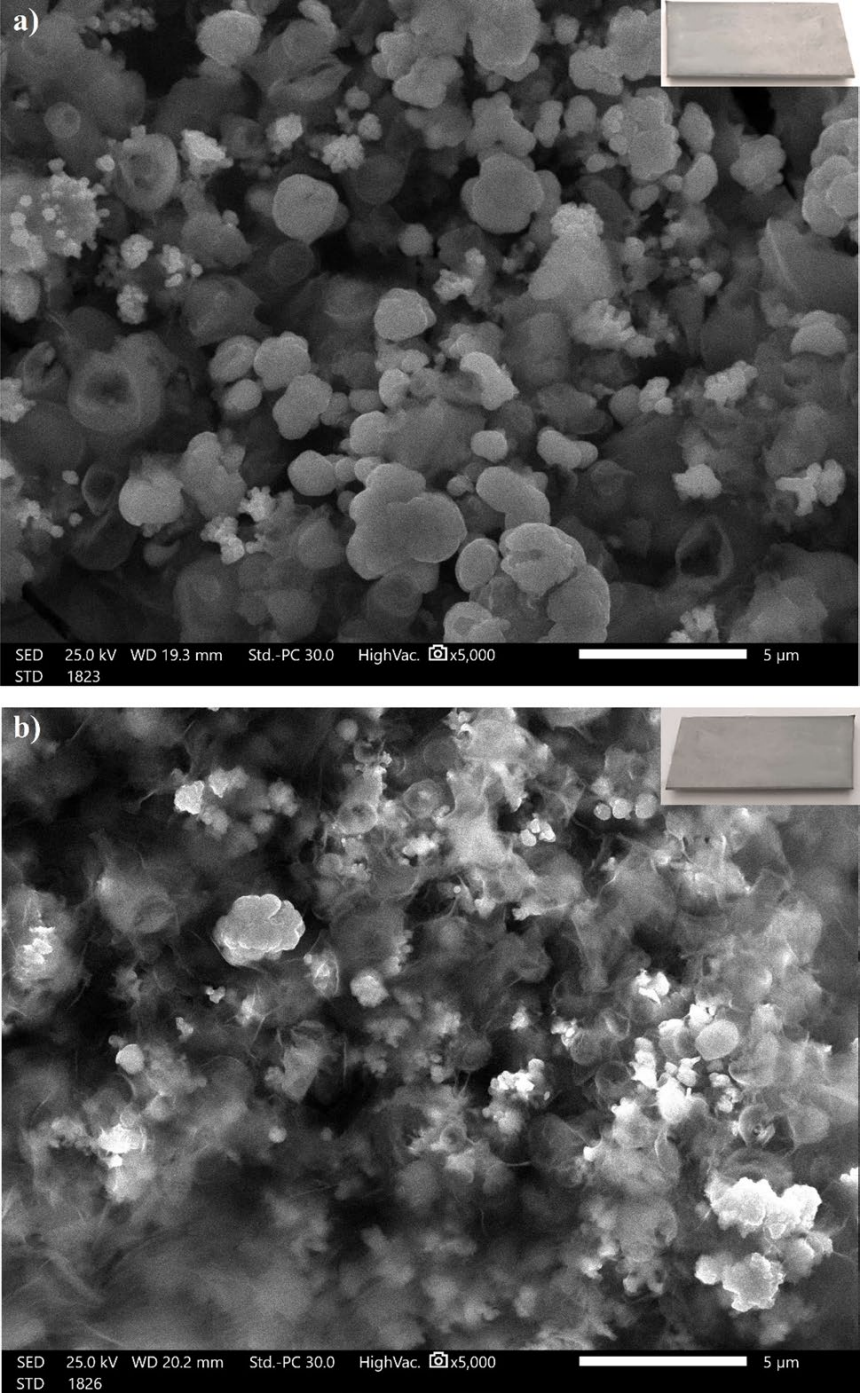
SEM micrographs of steel coated by (**a**) Ni-SA coating and (**b**) Ni-G-SA coating. The optical photographs of the coated steel are inserted as inset.

Surface morphology is an important factor to consider while studying superhydrophobic coatings. Figure 3a shows a SEM micrograph of steel grafted by Ni-SA coating, demonstrating that the deposited nickel possesses nano-sized circular-like particles. Some of the nano-sized particles aggregates to form larger particles. Figure 3b depicts a micrograph of steel grafted by Ni-G-SA coating; the figure illustrates that the deposited nickel coating has nano-sized circular-like particles that are smaller in size than Ni-SA coating. Obviously, graphene could serve as a nucleation site to improve the nucleation rate, so the Ni-G-SA coating has smaller nano-sized particles^51,52^. So, the Ni-G-SA has higher surface roughness and thus greater superhydrophobicity. The transparent flakes of graphene sheets can easily be seen. To determine the wettability behaviour of the prepared superhydrophobic coatings, CAs and sliding angles, SAs, were measured. The values of CAs for Ni-SA and Ni-G-SA coatings are 155.7° and 161.4°, while the values of SAs for both coatings are 4.0° and 1.0°, respectively. These results indicate that; the presence of graphene increases roughness and superhydrophobicity. The nano-micro structures can store air that can effectively hinder water from contacting the surface^53^.

### XRD results

**Figure 4:**
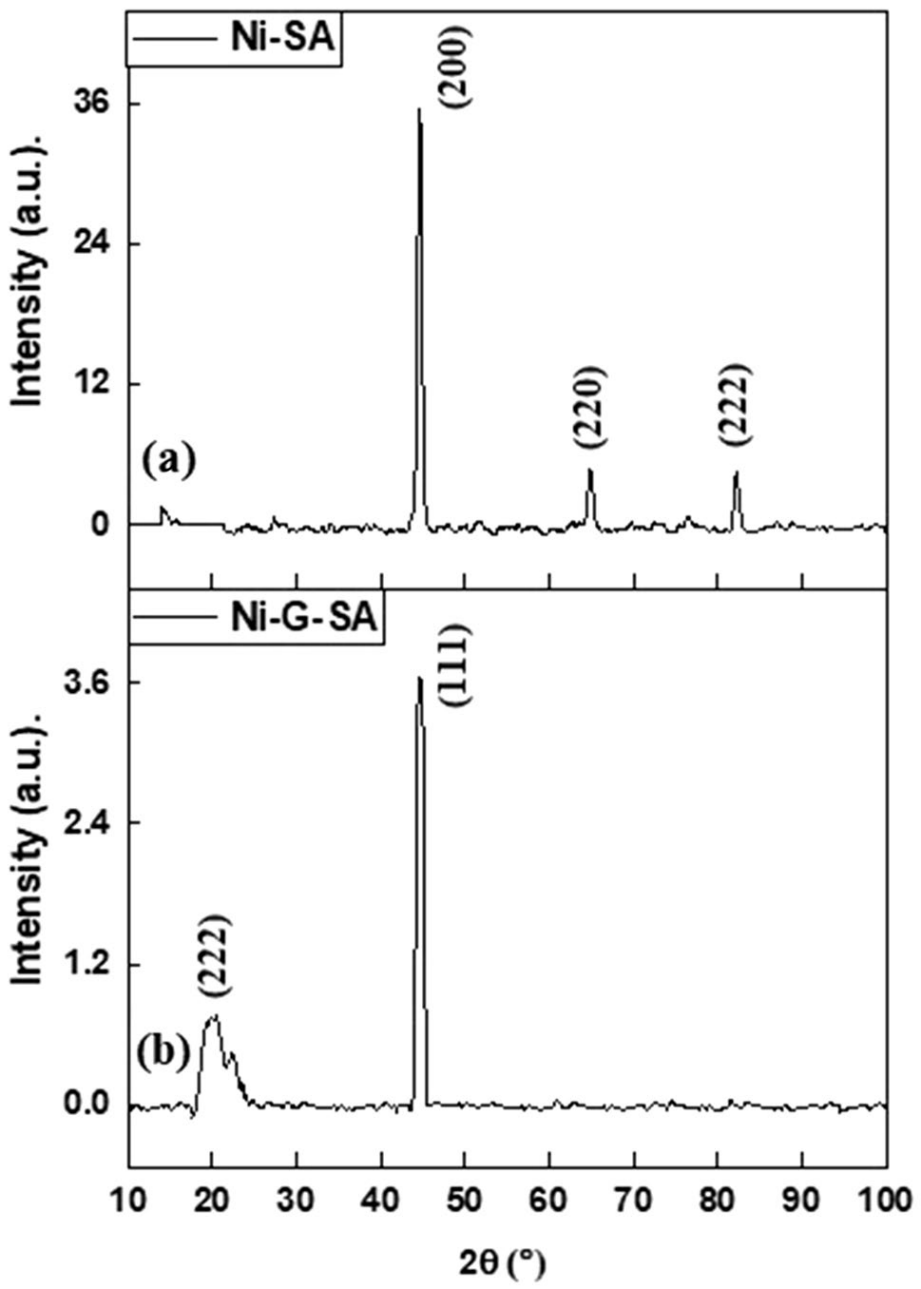
XRD patterns of steel coated by (**a**) Ni-SA coating and (**b**) Ni-G-SA coating.

The composition and crystal orientation of steel coated with Ni-SA and Ni-G-SA superhydrophobic coatings were determined using the XRD technique. Figure 4 depicts the XRD patterns of these coatings. For Ni-SA coating, there are three diffraction peaks at 2θ values of 44.6°, 64.7°, and 82.4° are, corresponding to faced cubic centered, fcc, of NiO (JCPDS card no. #47–1049). The (200) has the highest intensity of the three peaks, indicating that it is the preferred crystal orientation, with higher periodicity than the other orientations^54^. For Ni-G-SA coating, there are two diffraction peaks; the peak at 2θ values of 21.6° corresponds to graphene, while that at 44.5 corresponds to Ni^55^. The graphene peak is broad, showing that graphene has a small particle size. The NiO peaks are absent in the presence of graphene.

### Chemical stability

**Figure 5:**
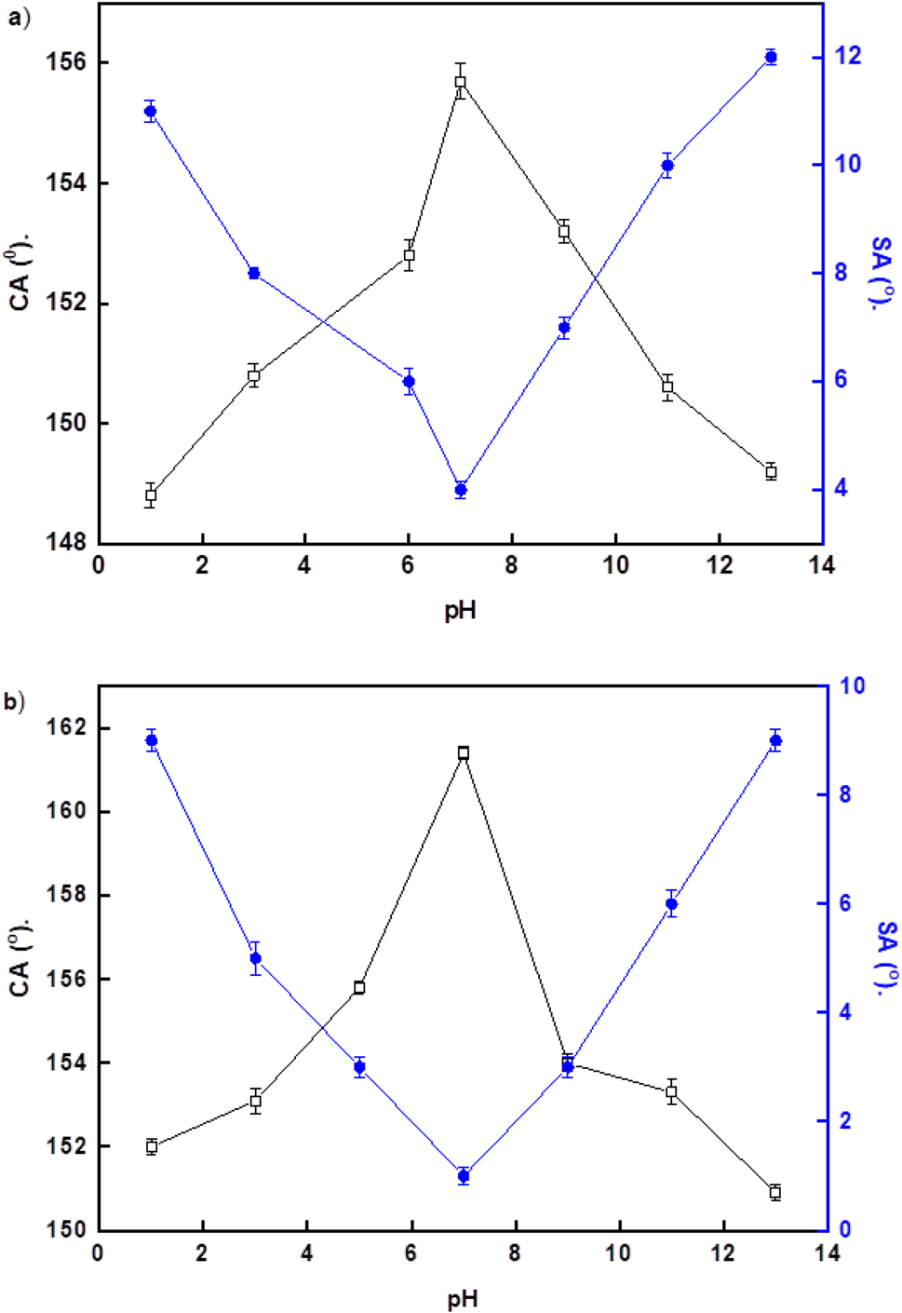
Variation of water droplets pH and their CAs and SAs on the steel coated by (**a**) Ni-SA coating and (**b**) Ni-G-SA coating.

Figure 5a,b depict the relationships between the CAs and SAs of water droplets on the prepared superhydrophobic surfaces and pH. The results indicate that Ni-SA coatings are superhydrophobic in the pH range of 3–11, whereas the Ni-G-SA coatings are superhydrophobic in the pH range of 1–13, where the CAs are frequently larger than 150°, and the SAs are less than 10°. As a result, nickel doping with graphene improves the chemical stability of the superhydrophobic coating in both basic and acidic conditions.

Table 2 summarizes the results of recent literature works on the chemical stability of the superhydrophobic surfaces on the steel substrate and their comparison with that of the prepared superhydrophobic coating in this study. As seen in the table, the prepared superhydrophobic coated steel has chemical stability superior to several previously recorded values.

**Table 2:** Comparison of the of the recently published research on the chemical stability of the superhydrophobic surfaces on the steel substrate with the prepared superhydrophobic surface.

Superhydrophobic coating	pH stability range
Ni/myristic acid^47^	3–11
TiO_2_/Zn/stearic acid^80^	2–12
Ni^81^	2–7
Cu-pentadecafluorooctanoic acid^82^	1–10
Lanthanum palmitate^83^	1–13
Ni-graphene/stearic acid (current work)	1–13

### Mechanical stability

**Figure 6:**
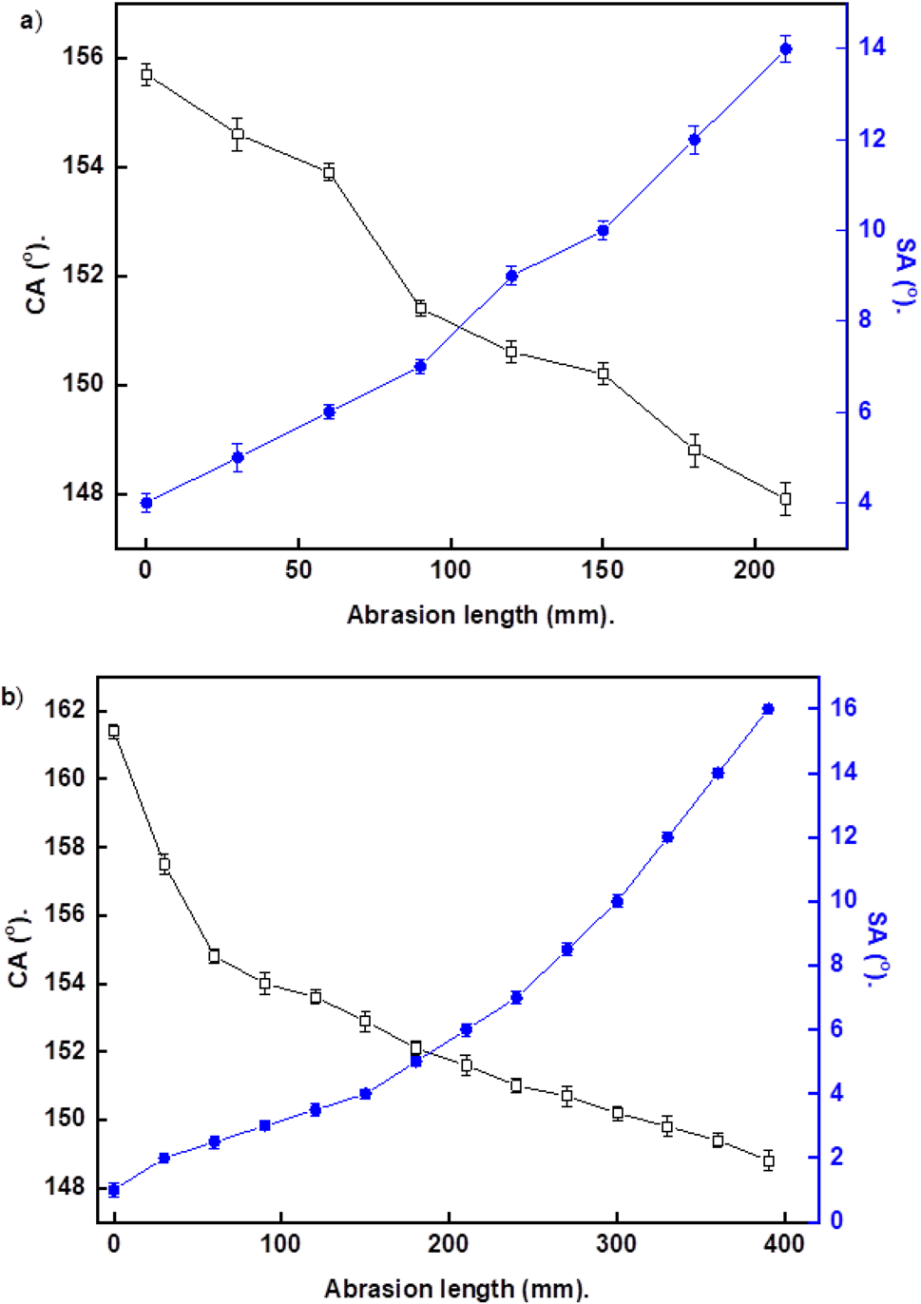
Variation of CAs and SAs with the abrasion length for steel coated by (**a**) Ni-SA coating and (**b**) Ni-G-SA coating.

In industrial applications, poor mechanical abrasion of manufactured superhydrophobic coatings is regarded as a major issue. Improving the abrasion resistance of superhydrophobic coatings has been identified as a critical aspect for their industrial applications^56^. Some superhydrophobic surfaces are even fragile to the finger contact^57^. Figure 6a,b demonstrate the relationships between contact and sliding angles of water droplets on prepared superhydrophobic coatings as a function of the abrasion length.

**Figure 7:**
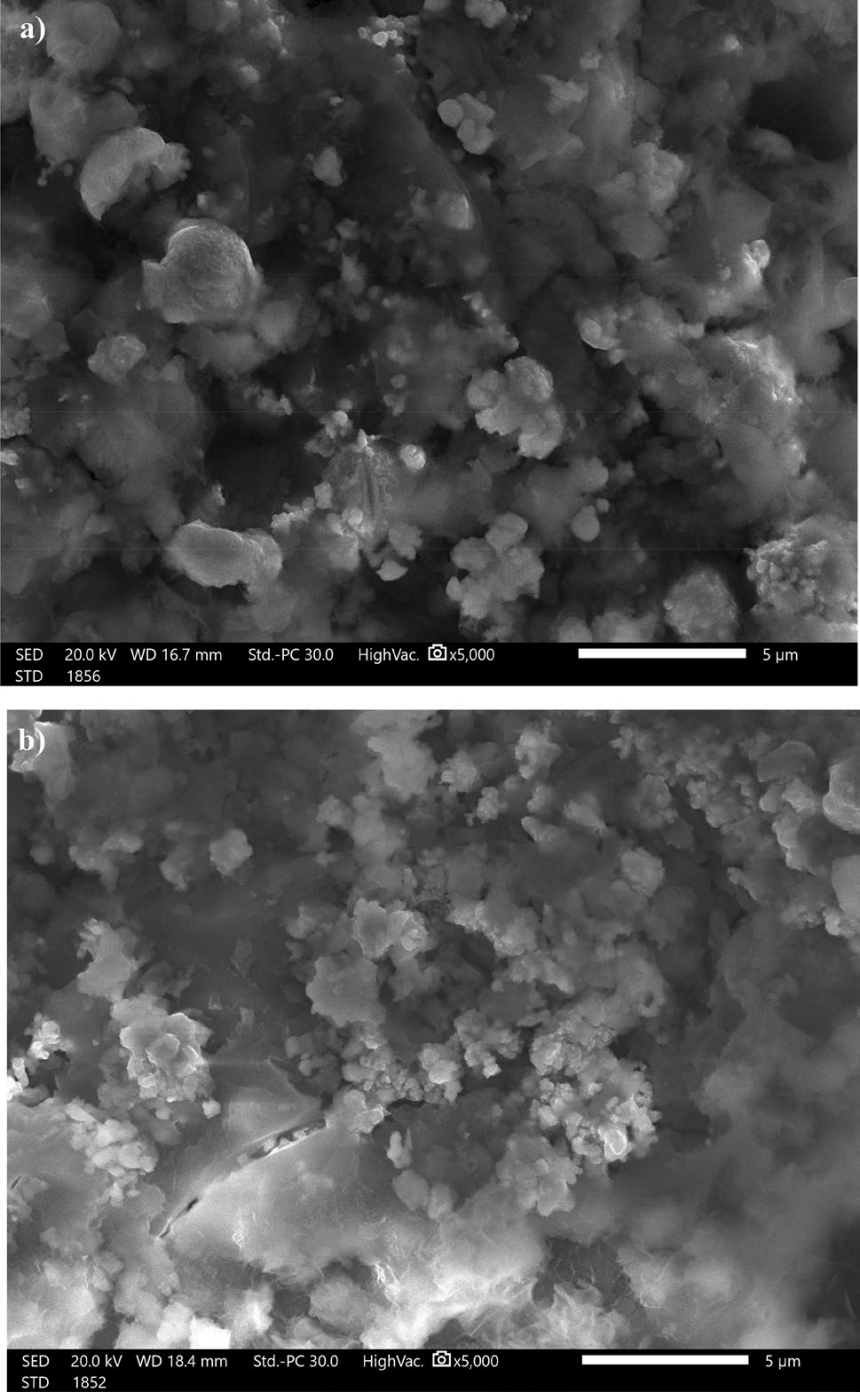
SEM images of steel coated by (**a**) Ni-SA coating and (**b**) Ni-G-SA coating after abrasion test.

The plots demonstrate that the CAs decreased, and the SAs increased as the abrasion length increased. The prepared superhydrophobic Ni-SA coating maintains its superhydrophobicity until an abrasion length of 150 mm. While the prepared superhydrophobic Ni-G-SA coating maintains its superhydrophobicity up to a 300 mm abrasion length. These results revealed that doping the prepared superhydrophobic Ni-SA coating with graphene producing Ni-G-SA significantly improves the mechanical stability. The enhanced mechanical resistance of steel coated with Ni-G-SA is related to the excellent tribological behaviors of graphene^58–61^. Figure 7 depicts the SEM micrographs of steel coated with Ni-SA and Ni-G-SA coatings after the abrasion test. The figure shows that the nano-sized circular-like particles were destroyed for the prepared coatings. Since the low surface energy and surface roughness are two critical requirements for superhydrophobic coating fabrication, so the destroying of the nano-sized circular-like particles roughness causes the manufactured coatings to lose their superhydrophobic properties.

Table 3 summarizes the findings of recent investigations on the mechanical abrasion resistance of superhydrophobic surfaces on steel substrates and their comparison to the produced superhydrophobic coating in this work. The prepared superhydrophobic coating is significant on the industrial scale as its good abrasion resistance, ease of the production procedure, as well as the availability and low cost of the chosen components.

**Table 3:** Comparison of the of the recently published work on the mechanical abrasion resistance of the superhydrophobic surfaces on the steel substrate with the prepared superhydrophobic surface.

Superhydrophobic coating	Abrasion pressure (kPa)/weight (g)	Sandpaper grit	Abrasion distance, mm
Mg(OH)_2_/STEARIC ACID^84^	100 g	1000	600
Cerium nitrate hexahydrate/myristic acid^75^	2.45 kPa	1000	600
Cu-1-octadecanethiol^57^	50 g	500	200
TiO_2_/Zn/stearic acid^80^	200 g	400	220
Zn/pentadecafluorooctanoic acid^85^	100 g	2000	100
Ni-graphene/stearic acid (current work)	3.00 kPa	800	300

### Corrosion resistance behaviour

#### Potentiodynamic polarization results

**Figure 8:**
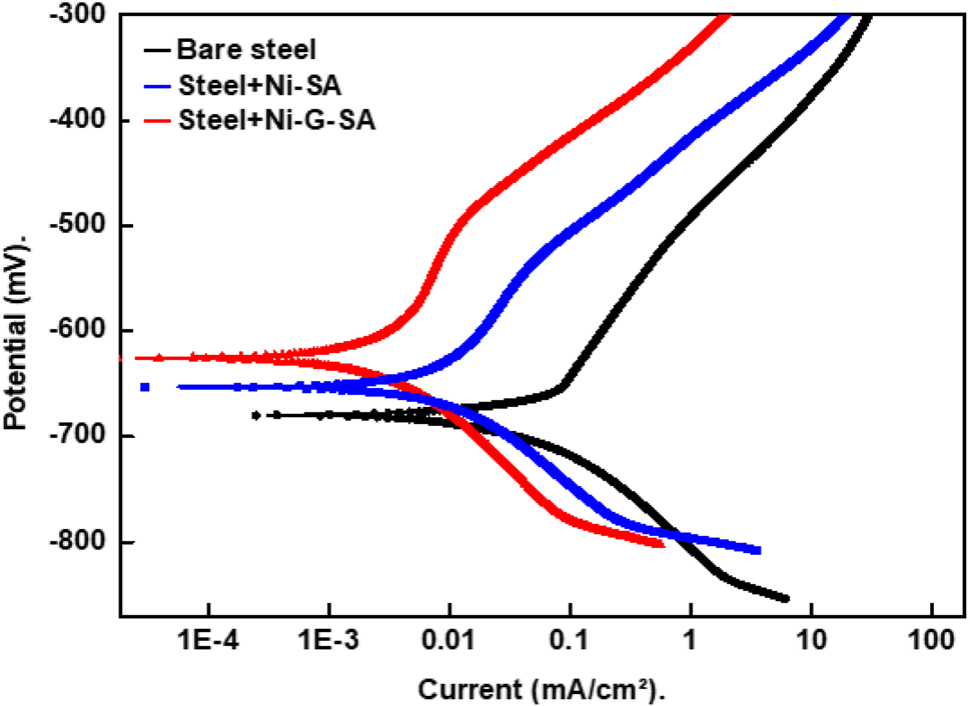
Potentiodynamic polarization curves for the bare steel and the superhydrophobic coated steel in 0.5 M NaCl solution.

The potentiodynamic polarization curves of bare steel and superhydrophobic coated steel by Ni-SA and Ni-G-SA in a 0.5 M NaCl aqueous solution are depicted in Fig. 8. The cathodic polarization curves are characterized by limiting diffusion currents, which are attributed to oxygen reduction reaction, Eq. (1).

1 $$ {\text{O}}_{{2}} + {\text{2H}}_{{2}} {\text{O}} + {\text{4e}} \to {\text{ 4OH}}^{ - } $$

Thus, the cathodic process is controlled by the oxygen gas diffusion from the bulk to the electrode surface. Table 4 depicts the potentiodynamic polarization parameters of the bare steel and superhydrophobic coated steel, including corrosion current density, i_corr._, corrosion potential, E_corr._, and protection efficiency, % P. Equation (2) was used to determine the protection efficiency^62^.

2 $$ \% {\text{P}} = \left[ {\left( {{\text{i}}_{{{\text{o}}.}} - {\text{ i}}_{.} /{\text{i}}_{{{\text{o}}.}} } \right)} \right] \times { 1}00 $$

**Table 4:** The potentiodynamic polarization parameters for the bare steel and the superhydrophobic coated steel in 0.5 M NaCl solution.

Deposit	− E_corr_, mV	β_a_, mV/decade	− β_c_, mV/decade	i_corr_, µA/cm^2^	%P
Bare steel	679	158	102	56.8	–
Steel + Ni-SA	651	155	89	5.6	90.1
Steel + Ni-G-SA	628	217	98	2.9	94.9

where, i_o._ and i are the corrosion current density for bare steel and superhydrophobic coated steel. The i_corr._ value for steel coated with Ni-SA is lower than that of bare steel, which can be related to the superhydrophobic behaviour of the coated steel. The trapped air around the superhydrophobic coating microstructures can reduce the contact area between the steel and the solution, resulting in a higher reduction in the i_corr_^63^. The presence of graphene improves the superhydrophobicity of the prepared Ni-G-SA coating, resulting in a greater decrease in the contact area of steel and the medium. So, the protection efficiency of steel coated by Ni-G-SA is higher than that of Ni-SA.

#### Electrochemical impedance spectroscopy results

**Figure 9:**
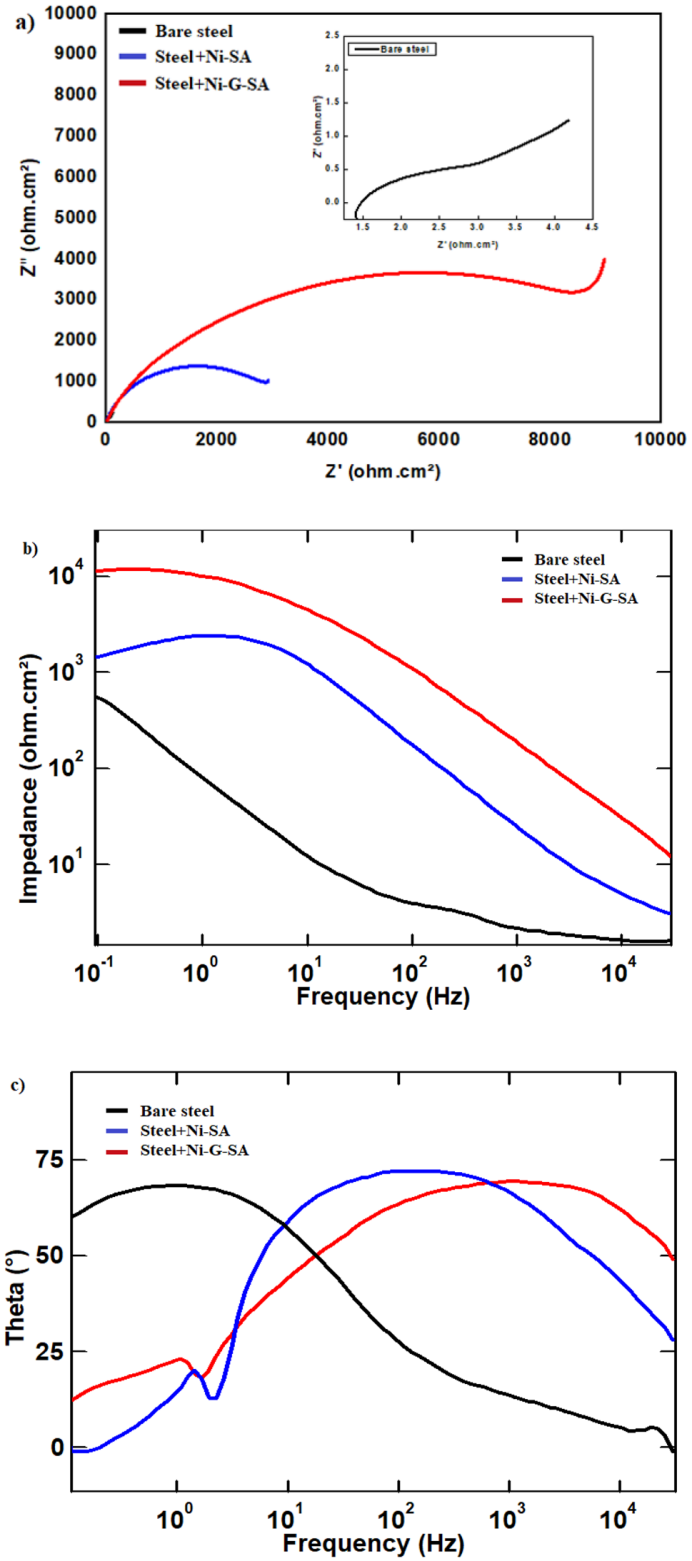
Nyquist and Bode plots of bare steel and superhydrophobic coated steel in 0.5 M NaCl solution.

The Nyquist and Bode plots of bare steel and superhydrophobic coated steel in 0.5 M NaCl solution are depicted in Fig. 9a–c. Nyquist plots, Fig. 9a, demonstrate a depressed capacitive semicircle, followed by a diffusion tail at low frequency. The depressed capacitive semicircle of the Nyquist plots at high frequencies is attributed to the interfacial charge transfer reaction^64^. The diffusion tail at low frequency is attributed to the diffusion process. Steel coated by Ni-G-SA shows the highest capacitive semicircle. The superhydrophobic coated steel blocks the active corrosion sites and limits the diffusion of the corrosive species, such as Cl^−^ and H_2_O, into the surface of steel metal.

According to Fig. 9b, the Bode plots for steel coated by Ni-G-SA in 0.5 M NaCl solution show the largest impedance magnitudes at the low frequency while the bare steel has the lowest value. This is attributed to the protective action of the prepared superhydrophobic coats on the steel substrate. The phase angle plot, Fig. 9c, shows two times constant at low and moderate frequencies for bare steel and coated steel surface. The time constant appearing at the low-frequency range was due to the protective superhydrophobic coating or the corrosion products in bare steel. The time constant appearing at the moderate or high frequency was attributed to the electrical double layer^65–67^.

**Figure 10:**
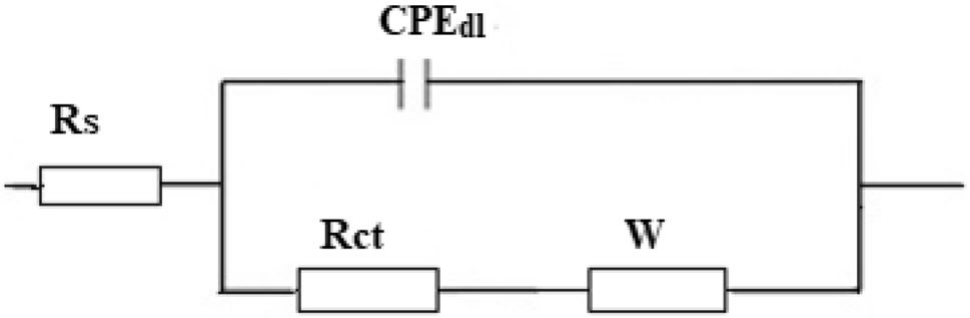
The equivalent circuit model.

The equivalent circuit shown in Fig. 10 was used to fit the EIS experimental data, and the impedance parameters were estimated by the Zsimpwin software. The equivalent circuit includes; solution resistance, R_s_, charge transfer resistance, R_ct_, double-layer constant phase element, CPE_dl_, and Warburg element. W. Table 5 depicts the EIS parameters of bare steel and superhydrophobic coated steel. The protection efficiency was determined using Eq. (3)^62^:

3 $$ \% {\text{P}}\, = \,[({\text{R}}_{{{\text{ct}}}} - {\text{  R}}_{{{\text{ct}}}}{^{{\text{o}}}} )/{\text{ R}}_{{{\text{ct}}}} ] \times {1}00 $$

**Table 5:** The impedance parameters for the bare steel and superhydrophobic coated steel in 0.5 M NaCl solution.

Deposit	Rs (Ω cm^2^)	n_1_	CPE_dl_ × 10^–6^ (s^n^ Ω^−1^ cm_2_)	W × 10^–4^	R_ct_ (Ω cm^2^)	%P
Bare steel	1.6	0.89	237.6	387.3	1.5	–
Steel + Ni-SA	2.9	0.86	20.60	6.924	3547	99.96
Steel + Ni-G-SA	4.4	0.79	5.300	1.238	8586	99.98

R_ct_^o^ and R_ct_ are the charge transfer resistance for the bare steel and superhydrophobic coated steel. Table 5 shows the obtained impedance parameters. Obviously, each of Rct and %P increase in the following order, bare steel < steel + Ni-SA < steel + Ni-G-SA, and so increasing the corrosion resistance in the same order.

Table 6 summarizes the results of recent literature studies on superhydrophobic coating corrosion resistance on the steel substrate and compares them to the corrosion resistance of the produced superhydrophobic coating in this investigation. The data in the table show that the prepared superhydrophobic coating has good corrosion resistance, so it has significant in the industrial sector.

**Table 6:** Comparison of the of the recently published investigations on the corrosion resistance of the superhydrophobic surfaces on the steel substrate with the prepared superhydrophobic surface.

Superhydrophobic coating	R_ct_ before treatment (Ω cm^2^)	R_ct_ after treatment (Ω cm^2^)	%P
Mg(OH)_2_/stearic acid^84^	848	2.87 × 10^5^	99.7
Ni/myristic acid^47^	135	6.50 × 10^3^	97.9
Fe_2_O_3_/stearic acid^86^	1.12 × 10^3^	10.80 × 10^3^	89.6
Ni-TiO_2_/tri ethoxy(octyl) silane^87^	480	14.96 × 10^4^	99.7
TiO_2_/Zn/stearic acid^80^	1.72 × 10^3^	1.63 × 10^4^	89.5
Ni-B_4_C/stearic acid^88^	1.506	162.40	99.1
Ni-Co-BN/fluoro alkyl silane^89^	5.76 × 10^3^	3.28 × 10^4^	82.5
Ni-Al_2_O_3_/stearic acid^90^	1.21 × 10^3^	8.20 × 10^3^	85.3
Zn/stearic acid^91^	3.97	3.13 × 10^3^	99.9
Dodecyl tri methoxy silane^92^	866	2.82 × 10^5^	99.7
Cerium nitrate hexahydrate/myristic acid^75^	6.96 × 10^5^	1.55 × 10^9^	99.9
Zn–Fe/myristic acid^93^	11.50	1.38 × 10^3^	99.2
Al_2_O_3_-γ-(2,3-epoxypropoxy) propyl tri methoxy sil-SiO_2_/steaic acid^94^	3.16 × 10^3^	3.09 × 10^4^	89.8
Tetra ethoxy silane-tri ethoxy silyl propyl isocyanate–aniline trimer^95^	10.50 × 10^4^	1.46 × 10^6^	92.8
Ni-graphene/stearic acid (current work)	1.50	8.59 × 10^3^	99.9

The enhanced corrosion resistance, chemical, and mechanical stability of the Ni-G-SA layer are due to its higher superhydrophobicity, the refined crystalline strengthening mechanism due to the small grain size of the nanostructures of the Ni-G-SA coating, the inclusion of graphene in a Ni matrix can effectively prevent dislocation sliding in the Ni matrix, the high chemical and mechanical stability, chemical inertness, impermeability, and hydrophobicity of graphene^34,52,68–74^. In Addition, it is established that graphene helps in preventing the oxidation of metal at the expense of its own oxidation.

#### Mechanism of anti-corrosion performance

The water molecules can be freely adsorbed to the bare steel surface. Chloride ions can also be adsorbed to the steel surface and form [FeClOH]^−^, causing severe corrosion of uncoated steel. As a result, water and Cl^−^ ions can easily contact the metal surface and start the corrosion process^75^.

The steel coated with superhydrophobic films, on the other hand, has a nanostructure that is covered by adsorbed hydrophobic material. Air can readily be trapped in the valleys between the rough surface's peaks. As a result of trapped air's obstructive influence, aggressive ion species such as Cl^−^ in the electrolyte or corrosive environment can hardly attack the underlying surface^18,75,76^. The air trapped on the superhydrophobic surface really works as a passivation barrier between the substrate and the corrosive environment. Furthermore, because the isoelectric point for superhydrophobic materials in neutral solutions was at pH 2–4, it was determined that the superhydrophobic surface in neutral solutions was negatively charged. The negative charge of a superhydrophobic surface resulted in a decrease in the concentration of Cl^−^ anion in the vicinity of a solid surface, which increased corrosion resistance^18^. It is reported that graphene has a negative zeta potential value due to the presence of electronegative functional groups formed at the graphite lattice^77–79^. So, the enhanced corrosion resistance of the steel coated with the superhydrophobic Ni-G-SA coating is due to its higher negative surface charge, so it has a lower concentration of Cl^−^ anion in the vicinity of a solid surface than the steel coated with Ni-SA coating.

## Conclusion

1. A high-quality graphene was prepared from an environmentally friendly biomass resource, rice straw.
2. A superhydrophobic Ni-SA and Ni-G-SA coatings were fabricated on the steel substrate.
3. The prepared superhydrophobic Ni-G-SA coating has a water contact angle of 161.4^o^, while the Ni-SA coating has a water contact angle of 155.7°. The presence of graphene improves the roughness of the prepared coat and so produces higher superhydrophobicity.
4. The chemical stability test indicates that the Ni-SA coating retains superhydrophobicity in the pH range 3–11, while the Ni-G-SA coating retains superhydrophobicity in the pH range 1–13.
5. The mechanical abrasion test showed that the prepared superhydrophobic Ni-SA coating exhibits superhydrophobicity until abrasion length of 150 mm; however, Ni-G-SA coating exhibits superhydrophobicity until abrasion length of 300 mm.
6. The presence of graphene in the prepared superhydrophobic coating improves its chemical and mechanical stability.
7. The potentiodynamic polarization results show that the corrosion current density values for the bare steel, steel coated by Ni-SA and Ni-G-SA in 0.5 M NaCl solution equal 0.057 mA/cm^2^, 0.0056 mA/cm^2^, and 0.0029 mA/cm^2^, respectively. The coating of steel with a superhydrophobic coating greatly decreases the corrosion current density, so the corrosion rate is greatly diminished. So, the doping of the superhydrophobic Ni-SA coating with graphene greatly improves the corrosion resistance behaviour. The electrochemical impedance spectroscopy results confirm the potentiodynamic polarization results.

## Data Availability

The datasets used and/or analysed during the current study available from the corresponding author on reasonable request.
